# Supplementary material for: Heterogenous Differences in Cellular Senescent Phenotypes in Pre-Eclampsia and IUGR following Quantitative Assessment of Multiple Biomarkers of Senescence
Source: Int J Mol Sci. 2023 Feb 4;24(4):3101. doi: 10.3390/ijms24043101 (PMC9963163; doi:10.3390/ijms24043101)
Supplement: Supplementary file 1 [file ijms-24-03101-s001.zip › ijms-2068439-supplementary.pdf]

Expression of senescence associated genes in Adverse pregnancy outcomes

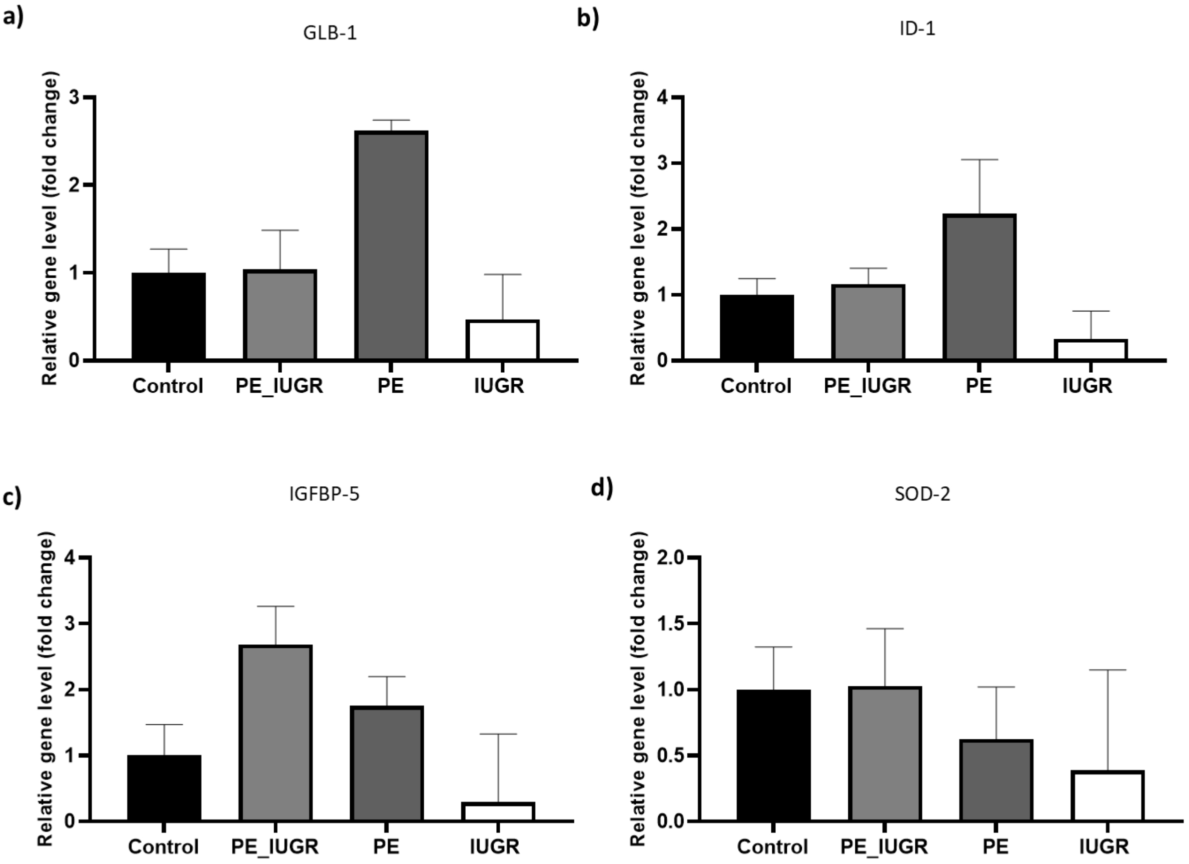

Supplementary Figure S1. Expression of senescence associated gene for (a) GLB-1, (b) ID-1, IGFBP-5 (c) and SOD-2 (d) showed no statistical differences when compared to controls.

Expression of nuclear intermediate filament protein Lamin B1 in placental tissue in Pre-eclampsia and IUGR

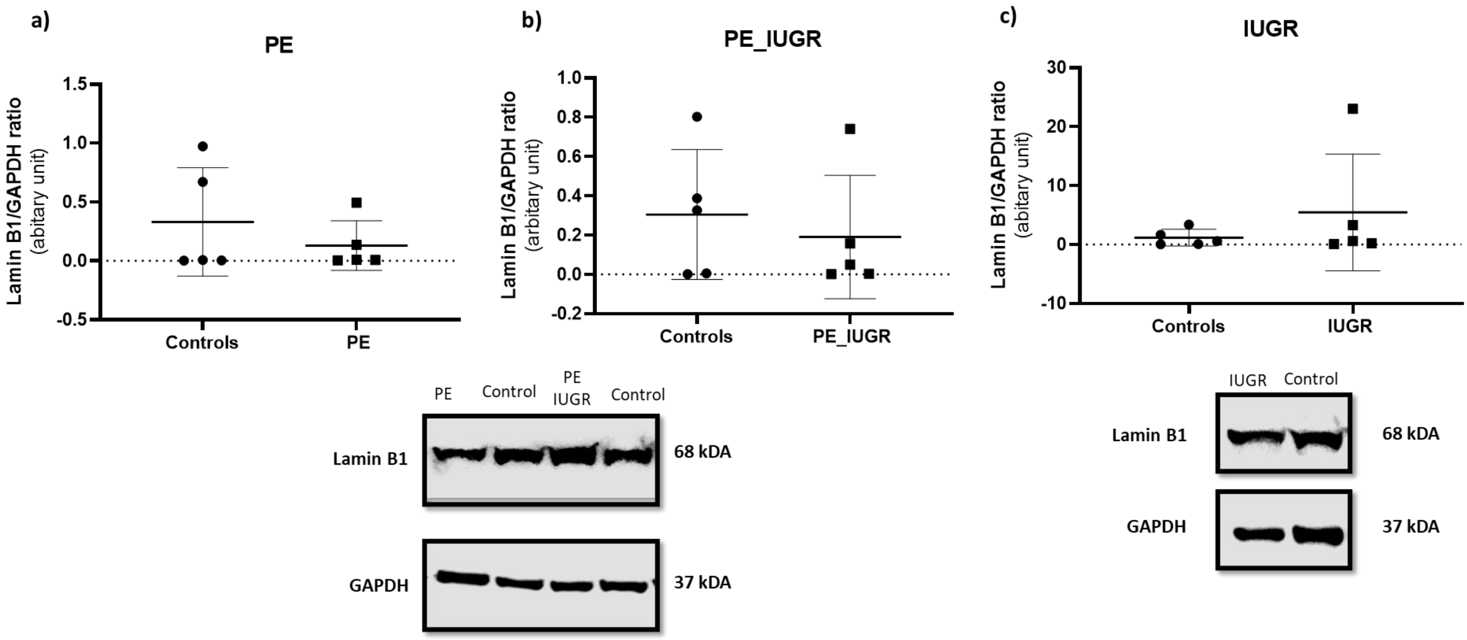

Supplementary Figure S2. Lamin B1protein expression in placental tissue for (a) PE only, n=5 (b) PE associated with IUGR, n=5 (c) IUGR <10th Centile, n=5. Statistical Analysis with Wilcoxon matched-pairs signed rank test with two-tailed test, v control placenta. Protein expression of Lamin B1 was normalized to the expression of the housekeeping reference protein GAPDH.

Absolute Quantification for mitochondrial DNA

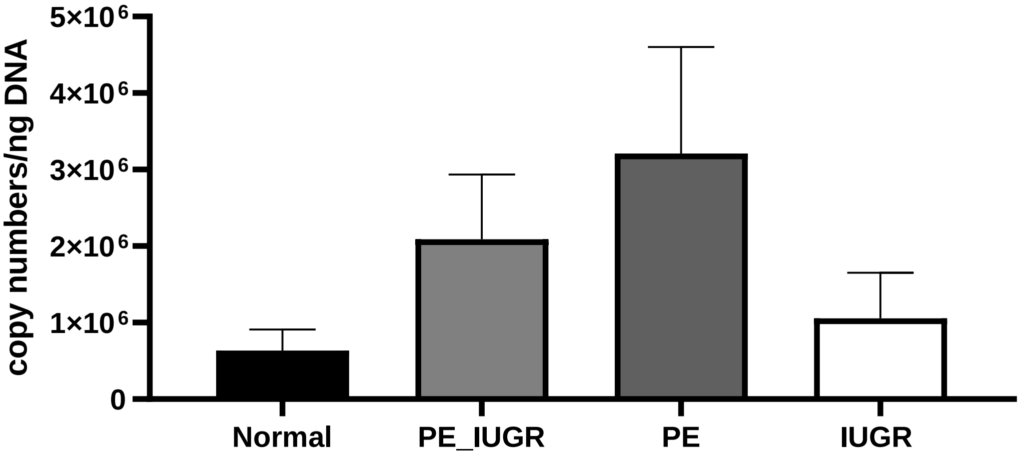

Supplementary Figure S3. Absolute quantification of mitochondrial DNA (*mtDNA*) shows no significant difference between cases and controls.
All cases showed elevated mtDNA copy numbers when compared to controls with PE (3211713 ± 2780530 v 634312 ± 1230023; P=0.058); PE IUGR (2089319 ± 2534706 v 634312 ± 1230023; P=0.19) and IUGR (1056158 ± 2144337 v 634312 ± 1230023; P=0.9)

Supplementary Table S1. Tata Binding Protein (TBP) Sybr green sequence:

TBP Forward Sequence	TGTATCCACAGTGAATCTTGGTTG
TBP Reverse Sequence	GGTTCGTGGCTCTCTTATCCTC
